# Soil Bacterial Diversity Is Positively Correlated with Decomposition Rates during Early Phases of Maize Litter Decomposition

**DOI:** 10.3390/microorganisms9020357

**Published:** 2021-02-11

**Authors:** Akane Chiba, Yoshitaka Uchida, Susanne Kublik, Gisle Vestergaard, Franz Buegger, Michael Schloter, Stefanie Schulz

**Affiliations:** 1 Research Faculty of Agriculture, Hokkaido University, Sapporo 060-8589, Japan; akane.chiba@helmholtz-muenchen.de (A.C.); uchiday@chem.agr.hokudai.ac.jp (Y.U.); 2 Research Unit Comparative Microbiome Analysis, Helmholtz Zentrum München, German Research Centre for Environmental Health, 85764 Neuherberg, Germany; susanne.kublik@helmholtz-muenchen.de (S.K.); gisves@dtu.dk (G.V.); schloter@helmholtz-muenchen.de (M.S.); 3 Crop Physiology, TUM School of Life Science, Technical University of Munich, 85354 Freising, Germany; 4 Section of Bioinformatics, Department of Health Technology, Technical University of Denmark, 2800 Kgs. Lyngby, Denmark; 5 Institute of Biochemical Plant Pathology, Helmholtz Zentrum München, German Research Centre for Environmental Health, 85764 Neuherberg, Germany; buegger@helmholtz-muenchen.de; 6 TUM School of Life Science, Technical University of Munich, 85354 Freising, Germany

**Keywords:** soil bacterial diversity, maize litter decomposition, Actinobacteria, *Caulobacteraceae*

## Abstract

This study aimed to investigate the effects of different levels of soil- and plant-associated bacterial diversity on the rates of litter decomposition, and bacterial community dynamics during its early phases. We performed an incubation experiment where soil bacterial diversity (but not abundance) was manipulated by autoclaving and reinoculation. Natural or autoclaved maize leaves were applied to the soils and incubated for 6 weeks. Bacterial diversity was assessed before and during litter decomposition using 16S rRNA gene metabarcoding. We found a positive correlation between litter decomposition rates and soil bacterial diversity. The soil with the highest bacterial diversity was dominated by oligotrophic bacteria including Acidobacteria, *Nitrospiraceae*, and *Gaiellaceae*, and its community composition did not change during the incubation. In the less diverse soils, those taxa were absent but were replaced by copiotrophic bacteria, such as *Caulobacteraceae* and *Beijerinckiaceae*, until the end of the incubation period. SourceTracker analysis revealed that litter-associated bacteria, such as *Beijerinckiaceae*, only became part of the bacterial communities in the less diverse soils. This suggests a pivotal role of oligotrophic bacteria during the early phases of litter decomposition and the predominance of copiotrophic bacteria at low diversity.

## 1. Introduction

Plant litter decomposition is important for the recycling of carbon (C) and other nutrients in soil. It not only increases the amount of soil organic C [1,2,3] but also enhances soil organic matter and drives aggregation [4,5]. The rates of litter decomposition depend on litter quality (i.e., C:N ratio) [6,7], climatic conditions [8,9], soil type [10,11], and soil- and plant-associated microbiomes [12,13]. As such, it is essential to understand these microbiomes as a driver for efficient litter decomposition.

Litter decomposition is a continuous process, marked by significant changes in the chemical composition of litter materials [14,15,16,17]. The early phase is initiated by the colonization of litter materials by soil microbes that benefit from soluble labile C sources (sugars and starch) [18,19]. This is succeeded by active microbial communities, increasing nutrient demands, such as nitrogen (N) and phosphorus (P) from other sources to maintain their cellular function and growth [20,21,22]. Thus, several studies have postulated that the interplay between copiotrophs, which preferentially consume labile C pools, and oligotrophs, which supply N and P by decomposing recalcitrant compounds, is important during the early phases of litter decomposition [23,24]. In this stage, bacterial community composition shifts are dynamic and closely correlated with the changes in the quantity and quality of C substrates [16,25]. During the later phases of litter decomposition, when labile C in the litter layer is depleted and lignin concentration increases, copiotrophs’ growth is reduced, and the litter decomposition processes are taken over by oligotrophic bacteria and diverse fungal communities as the major decomposers of recalcitrant C [26,27].

Although this general trend of bacterial succession during litter decomposition is well described, there is still a lack of knowledge regarding the impact of reduced soil and leaf bacterial diversity on the initial litter decomposition process. It is known that a large variety of soil bacterial species are able to produce diverse hydrolytic enzymes, catalyzing the early steps of litter decomposition. For example, beta-glucosidases are shared by multiple species belonging to different taxonomic groups, including the phyla Firmicutes and Proteobacteria [28,29]. This functional redundancy is important in maintaining functional stability in soil under different environmental conditions, including redox and soil nutrient statuses [30,31].

We postulate that (i) soil bacterial diversity is positively correlated with litter decomposition rates, even during early phases of litter decomposition, and despite the high availability of labile C. Moreover, it has been demonstrated that low bacterial diversity in soils increases the chance of exogenous species to establish themselves in that environment [32,33]. Hence, we further postulate that (ii) the invasion of litter-associated bacteria is more pronounced in soils with reduced bacterial diversity.

The impacts of microbial diversity and community shifts on ecosystem function have been studied in natural settings. For example, chronosequences of soil from the same parental material with different developmental stages (e.g., retreating glaciers) have been used as a model to assess correlations between microbial community composition and its function [34,35]. Furthermore, some authors focused on soils with different agricultural management history (e.g., monoculture vs. crop rotation) and investigated differences in functional patterns of soil microbial communities [36,37]. Others targeted sites with different plant species diversity, mainly to address the link between above- and below-ground biodiversity [38,39]. Those approaches have the advantage of taking place under natural conditions. However, in many cases, reduced diversity is associated with shifts in abiotic soil properties, which mask the effect of microbial diversity on ecosystem function. Alternatively, microbial diversity can be manipulated in a defined manner. To reduce or eliminate microbes, soils are treated with chloroform fumigation [40], autoclaving [41], or gamma radiation [30,42]. The magnitude of the microbial loss differs depending on sterilization methods (e.g., the number of autoclaving cycles) [43] and incubation conditions after sterilization (e.g., the length of the preincubation period) [44]. Many authors used such sterile soils as substrates and inoculated microbial communities with different diversity levels [30,41,42].

**Figure 1:**
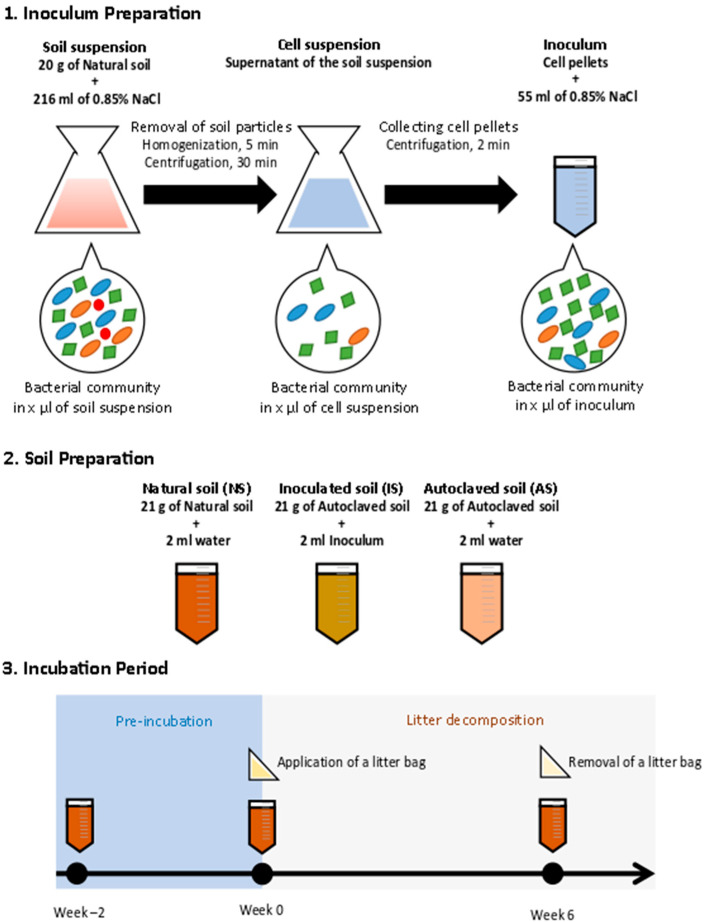
Schematic diagram of the experimental setup for the preparation of microbial inoculum and soils, and the incubation period.

In this study, we performed a litter decomposition experiment under controlled conditions using natural and autoclaved maize leaves. We compared the litter decomposition rates in soils with different levels of bacterial diversity, prepared from one natural soil (NS) by manipulating its bacterial diversity. Autoclaving and reinoculation with an extract of microbes from NS eliminated parts of the “rare biosphere”. This approach substantially reduced bacterial diversity in autoclaved soil (AS) and inoculated soil (IS) compared to NS (Figure 1), while no significant differences in bacterial abundance were determined among the soil treatments after a preincubation period. Thus, our data highlight the effect of soil bacterial diversity on its function (litter decomposition rates). We aimed to understand the role of bacterial diversity, mainly during the early phase of litter decomposition. Thus, litter materials were incubated in soil treated in three different ways for 6 weeks. To ensure that the diversity but not the abundance of bacteria differed among the soil treatments, the litter materials were added after 2 weeks of the preincubation period, allowing bacterial abundance to recover. Litter decomposition rates, bacterial community composition, and abundance were analyzed for 6 weeks. Our study highlighted the importance of soil bacterial diversity during the early phase of litter decomposition, especially with the presence of both copiotrophic and oligotrophic bacteria.

## 2. Materials and Methods

### 2.1. Sampling

Soil and maize (*Zea mays* L.) leaf samples were obtained from an agricultural field in Scheyern, Germany (latitude 48°29′51″ N, longitude 11°26′39″ E) in October 2015. The soil was characterized as Luvisol with an organic C content of 17.5 g C kg^−1^ dry soil, a total N content of 1.75 g N kg^−1^ dry soil, and a silt loam texture consisting of 18.9% sand, 58.6% silt, and 22.5% clay and a pH of 6.0. The soil was sieved through a 5 mm sieve. The natural and autoclaved maize leaves had an organic C content of 460 g C kg^−1^ dry matter and a total N content of 13.0 g N kg^−1^ dry matter.

### 2.2. Incubation Experimental Design

Soils with different levels of bacterial diversity (NS, IS, and AS) were prepared from one source as depicted in Figure 1. To prepare AS and IS, approximately 800 g of NS was placed in a 1 L glass bottle and autoclaved at 134 °C for 2 h [45]. After gently homogenizing the autoclaved soil, the bottle was kept at room temperature with the cap loosened to remove headspace moisture, to prevent airborne contamination. Another run of autoclaving was carried out after 2 days, followed by moisture removal. The autoclaved soil was stored at 4 °C until further use. We then checked for live copiotrophic bacteria by plating soil extracts on Luria-Bertani (LB) agar plates. To further check for a general reduction of active microbes, RNA was extracted from the soils using the protocol described by Töwe et al. [46] and quantified with the Quant-iT RiboGreen RNA Assay Kit (Thermo Fisher Scientific, Darmstadt, Germany). The results showed very little amounts of RNA (<0.30 ng g^−1^ dry soil, Table S1).

The inoculum for IS was prepared by isolating microbial fractions from NS according to Bressan et al. [47] with a small modification of a centrifugation speed. Briefly, 20 g of NS was suspended in 216 mL of 0.85% NaCl solution. After homogenizing for 5 min, the suspension was centrifuged at 100× *g* at 4 °C for 30 min to remove large soil particles. The supernatant was transferred to a new tube and centrifuged at 10,000× *g* for 2 min to obtain a cell pellet. The pellet was resuspended in 55 mL of 0.85% NaCl solution. In total, 2 mL of the resultant bacterial suspension, equivalent to 2.38 × 10^7^ copies of the 16S rRNA gene, was added to 21 g of AS, resulting in “IS” inoculated soil. To control for volume, 2 mL of distilled water was added to 21 g of NS and AS instead of the bacterial suspension.

For the incubation experiment, the 21 g of NS, AS, and IS were placed in Falcon^TM^ 50 mL conical centrifuge tubes (Corning, Kaiserslautern, Germany) with open headspace. The tubes were loosely covered by aluminum foil during the incubation period to avoid airborne contamination. Soil moisture in all treatments was adjusted to 60% of field capacity and maintained by adding distilled water throughout the experiment. Temperature was set to 18 °C and incubation was carried out in the dark. In order to allow the inoculated microbial community to adapt to the soils and reach a similar bacterial abundance as in NS based on 16S rRNA gene copies g^−1^ dry soil, all the soil samples were preincubated for 2 weeks (from week −2 to week 0). The length of the preincubation period was determined in a preliminary experiment for 3 weeks, where bacterial abundance based on 16S rRNA gene copies g^−1^ dry soil and diversity based on terminal restriction fragment length polymorphism (T-RFLP) profiles of 16S rRNA gene were tested every week (Text S1: Experimental design for the preliminary test). Accordingly, the preincubation period was set for 2 weeks (Figure S1).

After preincubation, autoclaved litterbags (53 µm-nylon mesh bags), filled with natural or autoclaved maize litter (0.5 g each, cut into 5 × 5 mm pieces), were placed into the soil at a depth of about 1 cm. For autoclaved maize litter, a part of natural maize litter was autoclaved for 20 min at 121 °C and stored at 4 °C until use. Sterility of the autoclaved maize litter was proven by putting pieces of the autoclaved maize litter on LB agar plates and subsequent incubation for 14 days at 20 and 37 °C. No microbial colonies were observed on the plates after the incubation, while the presence of slow-growing microbes might not be excluded completely.

Soil samples were taken after the addition of the inoculum (week −2), after preincubation (week 0), and 6 weeks after incubation with the litter materials (week 6), and immediately frozen at −20 °C for molecular analysis. For each treatment and time point, three parallel tubes were prepared and treated as true replicates throughout the experiment. Litter materials were sampled on weeks 0 and 6. An aliquot of the litter material was dried at 60 °C for >48 h to obtain dry weight, and the remaining was stored at −20 °C. Litter decomposition rates were assessed by calculating the quotient of remaining and initial litter amounts (dry weight basis). Due to the very small amount of litter materials left in the litter bag on week 6, further molecular analysis was performed only on the maize samples collected on week 0.

### 2.3. DNA Extraction and Library Preparation

Genomic DNA was extracted from 0.30 g of soil and 0.01 g of maize litter by using the NucleoSpin^®^Soil Kit (Macherey-Nagel, Düren, Germany). For cell lysis of soil and plant-associated microbes, buffer SL2 was used. As an extraction control (blank), an extraction without soil or maize litter was also processed. For the absolute quantification of bacteria and fungi in the soil and litter materials, 16S rRNA gene and internal transcribed spacer (ITS) regions were targeted as a proxy for bacterial and fungal abundance, respectively. SYBR Green-based quantitative real-time PCR (qPCR) assays were performed on a 7300 Real-time PCR System (Thermo Fisher Scientific, Darmstadt, Germany) with the primer pairs FP16S and RP16S primers for bacteria [48] and ITS1 and ITS4 primers for fungi [49], respectively (Table S2). The 16S rRNA gene from *Pseudomonas putida* and ITS from *Trichoderma reesei*, which were cloned into pCR^®^-Blunt Vector (Zero Blunt™ PCR Cloning Kit, Thermo Fisher Scientific, Darmstadt, Germany), were used as a standard. The optimal dilution rate of DNA extracts was 1:32 for both soil and maize litter samples. PCR reaction mixtures (25 μL) contained 12.5 μL of Sybr Green PCR Master Mix (Thermo Fisher Scientific, Darmstadt, Germany), 10 pmol of each primer, 8.5 μL of DEPC treated water, and 2 μg of DNA template. Amplification program of 16S rRNA gene was initiated by a denaturation step at 95 °C for 10 min, followed by 35 cycles of 95 °C for 20 s, 60 °C for 1 min, and 72 °C for 30 s, while that of ITS began with a denaturation step at 95 °C for 10 min, followed by 40 cycles of 94 °C for 30 s, 53 °C for 30 s, and 72 °C for 45 s A melting curve analysis was performed in a final cycle of 95 °C for 15 s, 60 °C for 30 s, and 95 °C for 15 s. The amplification efficiency was calculated from the formula Eff = [10^(−1/slope)^ − 1] × 100. Amplification efficiencies of qPCR runs in this study exceeded 80% and R^2^ value exceeded 0.99.

For the assessment of bacterial communities and diversity, the “16S Metagenomic Sequencing Library Preparation” protocol (Illumina, San Diego, CA, USA) and quality guidelines recommended by Schöler et al. [50] were used. DNA extracts were amplified in triplicate using the primer S-D-Bact-0008-a-S-16 and the primer S-D-Bact-0343-a-A-15 [51] (Table S2). PCR reaction mixtures contained 1 µL of template DNA, extraction blank or nuclease-free water (negative controls), 0.5 µL of 10 pmol of each primer, 1.5 µL of 3% BSA, 12.5 µL of NEBNext High-Fidelity 2X PCR Master Mix (New England Biolabs, Frankfurt am Main, Germany), and 8 µL of DEPC-treated water. The amplification program for the 16S rRNA gene was initiated at 98 °C for 5 min, followed by 30 cycles of 98 °C for 10 s, 60 °C for 30 s, and 72 °C for 30 s, and terminated at 72 °C for 5 min. The success of the PCR was checked on a 1.5% Agarose gel. The PCR triplicates were combined and purified using Agencourt AMPure beads (AMPure/PCR product ratio = 0.8) (Beckman Coulter, Krefeld, Germany). The length of the amplicon fragments was assessed using the BioAnalyzer 2100 instrument (Agilent Technologies, Santa Clara, CA, USA ) using a DNA 7500 chip (Agilent Technologies, Santa Clara, CA, USA). The amplicons were quantified with the Quant-iT PicoGreen dsDNA Assay Kit (Thermo Fisher Scientific, Darmstadt, Germany). Indexing PCR was performed in a reaction mix (25 µL) consisting of 10 ng of the purified amplicons, 2.5 µL of each indexing primer (Nextera^®^ XT Index Kit v2 set A; Illumina, San Diego, CA, USA), 12.5 µL NEBNext High-Fidelity 2X PCR Master Mix, and 6.5 µL DEPC treated water. Afterward, all amplicons were purified using Agencourt AMPure beads (AMPure/PCR product ratio = 0.8) and quantified with the Quant-iT PicoGreen dsDNA Assay Kit. Their quality was checked using the Bioanalyzer 2100. Extraction and PCR controls did not give any amplification products at the end of the library preparation procedure. Each library was diluted to 4 nM and sequenced with the MiSeq Reagent kit v3 (600 cycles) (Illumina, San Diego, CA, USA) for paired-end sequencing. Sequences were deposited in the NCBI Sequence Read Archive and are available under the accession numbers SRP127524.

### 2.4. Statistics and Bioinformatics

Raw sequence data were separated from their adapters using AdapterRemoval v2.1.0, and reads were trimmed with a Phred score of 15 and a minimum read length of 50 bp [52]. Datasets were subsequently analyzed using the QIIME 2 software package v2018.8.0 [53]. For quality control and trimming, 10 bp from N-terminus were truncated and the reads from C-terminus at positions 220 bp (forward) and 160 bp (reverse) were removed using the QIIME 2 plugin DADA2 v1.3.4 [54], followed by merging the paired reads and removing chimeric sequences with default filtering parameters. The generated unique amplicon sequence variants (ASVs) were used for taxonomic assignment using the SILVA database (release 132) [55] trained with a Naïve Bayes classifier [56]. Sequences assigned to chloroplasts at the order level and singletons were removed before further analyses. The dataset was rarefied to 33,899 reads per sample, reflecting the lowest number of the obtained reads per sample.

Statistical analyses and data visualization were carried out using the statistical software R v3.5.3 [57] and RStudio v1.1.463 [58]. One-way analysis of variance (ANOVA) was performed using the function aov to determine the effect of maize treatments and soil treatments on the mass loss of natural and autoclaved maize litter after 6 weeks, followed by a pairwise t-test and the Benjamini–Hochberg adjustment of *p*-values [59] using the function pairwise.t.test in the R Stats package. Two-way ANOVA was performed using the function aov to test the effect of time points, soil treatments, and the interaction between time points and soil treatments on bacterial and fungal abundance, and alpha-diversity indices, followed by Tukey’s HSD post-hoc test with a significance level of 0.05 using the function TukeyHSD. Bacterial and fungal abundance were log-transformed before statistical tests to improve the normality of the data distribution. Alpha-diversity of soil bacterial communities was measured by the Shannon–Weaver diversity index, the Pielou’s evenness, the number of observed ASVs using the vegan package v2.5-5 [60]. Variation in bacterial communities among the samples was displayed in nonmetric multidimensional scaling (NMDS) of Bray–Curtis dissimilarity using the function metaMDS in the vegan package. To test the effects of soil treatments and time points on the bacterial community dissimilarities, we conducted two-way permutational analysis of variance (PERMANOVA [61]) using the function adonis in the vegan package. Bacterial community compositions at the family level and categories (“Common”, “Accessory”, and “Unique”) were displayed in pie charts using the ggplots2 package [62]. Bacterial families existing across all the soils were defined as “Common”, those unique to a soil were represented as “Unique”, and the rest were grouped as “Accessory”. “Common;Others”, “Unique;Others”, and “Accessory;Others” contained common/unique/accessory bacterial families that were less than 2% in relative abundance. Changes in the relative abundance of dominant bacterial families (average relative abundance within three replicates >2%) across time points and soil treatments were tested using two-way ANOVA, followed by the Benjamini–Hochberg adjustment of *p*-values. The effect of soil treatments on the relative abundance was determined by performing pairwise comparisons of the least-squares (LS) means among the soil treatments at each time point using the function lsmeans in the lsmeans package [63]. To evaluate the correlation between litter decomposition rates and soil bacterial diversity indices, Pearson’s correlation coefficients were measured using the function cor.test in the stats package. SourceTracker was used to determine the origin of ASVs in the soils on week 6 [64]. Maize and soil samples on week 0 were considered as “source”, while soils on week 6 were considered as “sink”. The ASVs that did not match a specific source environment were marked “unknown”.

## 3. Results

### 3.1. Litter Decomposition 

The decomposition rates of natural maize litter were significantly higher in NS compared to IS and AS after 6 weeks of incubation, corresponding to 66.7%, 44.6%, and 37.8% of the litter mass loss in NS, IS, and AS, respectively (Figure S2). Differences in litter decomposition rates between autoclaved and natural maize litter were not significant for any soil treatments.

### 3.2. Dynamics of Bacterial and Fungal Abundance

**Figure 2:**
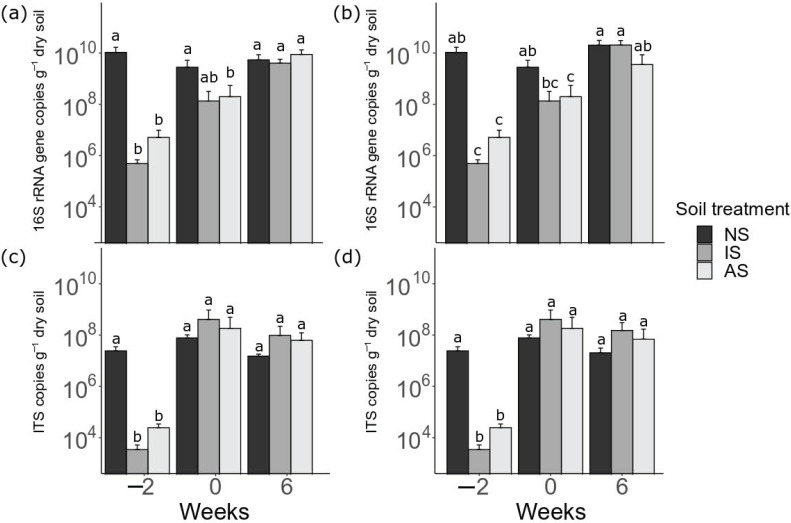
Copy numbers of the 16S rRNA gene and ITS regions representing bacterial and fungal abundance in soils (NS: natural soil, IS: inoculated soil, and AS: autoclaved soil) receiving natural (**a**,**c**) or autoclaved maize litter (**b**,**d**). Values represent the mean and the standard deviation of three replicates. Soils were incubated for 2 weeks (from week −2 to 0) and then received litter materials (week 0). The final sampling took place after 6 weeks of litter incubation (week 6). Bacterial and fungal abundance were log-transformed before statistical tests to improve the normality of the data distribution. We performed a two-way ANOVA to investigate the interaction between time points and soil treatments on bacterial and fungal abundance, followed by Tukey’s HSD test for pairwise comparisons. Different letters indicate significant differences (*p* < 0.05) according to Tukey’s HSD test.

The effect of soil treatments on bacterial abundance was dependent on the time points (Figure 2: *p* < 0.05, two-way ANOVA). On week −2, bacterial abundance in IS and AS was significantly lower compared to NS (*p* < 0.05, Tukey’s HSD test). Bacterial abundance in IS and AS increased from week −2 to 0, while there were no changes in bacterial abundance in NS during the same period. As a result, no significant difference in bacterial abundance between NS and IS was observed on week 0. Bacterial abundance in AS was still lower than in NS at the same time point (*p* < 0.05, Tukey’s HSD test). In general, the application of maize litter introduced a significant increase in bacterial abundance for AS and IS, which resulted in no differences in bacterial abundance among NS, IS, and AS on week 6, irrespective of the maize treatments (6.10 × 10^9^ and 1.48 × 10^10^ copies g^−1^ dry soil when averaged across the soil treatments receiving natural and autoclaved maize litter, respectively).

Similarly, the effect of soil treatments on fungal abundance was dependent on time points (*p* < 0.05, two-way ANOVA). On week −2, fungal abundance was significantly lower in IS and AS than NS (*p* < 0.05, Tukey’s HSD test). During the preincubation period, the fungal abundance of IS and AS increased while no changes were observed in NS. As a result, no significant differences in fungal abundance were observed among the soil treatments on week 0. The fungal abundance remained static until the end of the incubation experiment (8.03 × 10^7^ and 5.84 × 10^7^ copies g^−1^ dry soil when averaged across the soil treatments receiving natural and autoclaved maize litter, respectively).

### 3.3. Development of Bacterial Communities over Time 

A total of 4,224,002 raw reads were obtained. After filtering, 1,481,958 reads remained. The number of reads obtained from sequencing and after filtering is summarized in Table S3. The dataset was rarefied to 33,899 reads per sample, reflecting the lowest read number per sample (IS, week −2, replicate 1). Rarefaction curves indicated that sampling depths were sufficient as curves flattened for all samples (Figure S3). As a result, 10,558 ASVs were produced and assigned to 24 different phyla and 442 different families.

#### 3.3.1. Soil Bacteria

Alpha-diversity based on Shannon–Weaver diversity index, Pielou’s evenness, and the number of ASVs, is summarized in Table 1. Both, soil treatments and time points, had a major impact on the assessed indices (*p* < 0.05 for each factor, two-way ANOVA). NS had higher values than IS and AS throughout the experiment (*p* < 0.05, Tukey’s HSD test), while no difference was observed between IS and AS until the end of the incubation.

**Figure 3:**
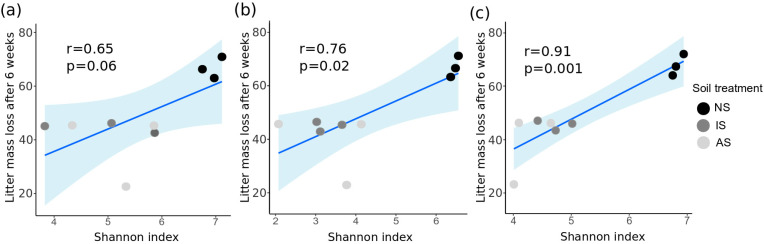
Scatter plots and regression lines showing the correlation between litter mass loss after 6 weeks and Shannon–Weaver diversity index on week −2 (**a**), week 0 (**b**), and week 6 (**c**). Additionally, the 95% confidence interval, the Pearson’s correlation coefficient (r), and *p*-value (*p*) are displayed. Black, gray, and light gray dots indicate different soil treatments (NS: natural soil, IS: inoculated soil, and AS: autoclaved soil, n = 3).

Pearson’s correlation analysis between litter mass loss after 6 weeks of incubation and alpha-diversity indices of the soil bacterial communities on week 0 revealed a significant (*p* < 0.05) and positive correlation (Figure 3 and Figure S4). Pearson’s correlation coefficient was 0.76, 0.80, and 0.68 for the correlation of litter mass loss and Shannon–Weaver diversity index, Pielou’s evenness, and number of ASVs, respectively. Interestingly, this correlation became stronger when leaf litter decomposition was linked to alpha-diversity indices on week 6, but was not significant on week −2.

**Figure 4:**
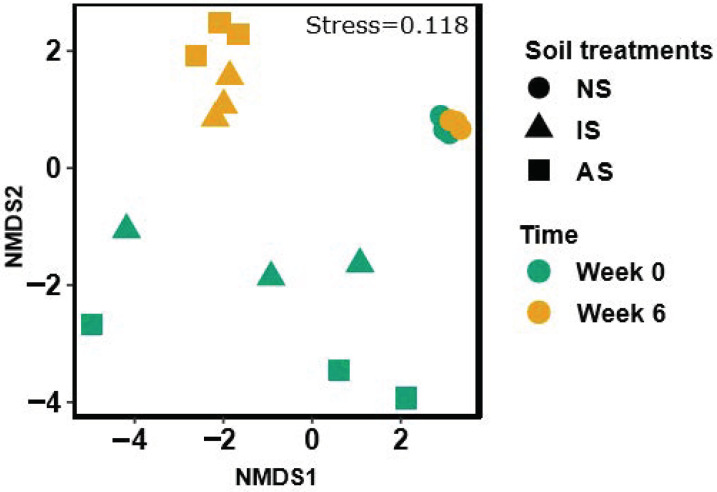
Variations in bacterial communities among soil samples (NS: natural soil, IS: inoculated soil, and AS: autoclaved soil) with natural maize litter displayed in nonmetric multidimensional scaling (NMDS) of Bray–Curtis dissimilarity (n = 3). Circle, triangle, and square shapes stand for NS, IS, and AS. Green and yellow colors represent the time points when maize litter was applied (week 0) and 6 weeks after the litter application, respectively.

Variations in beta-diversity of the bacterial communities are displayed in Figure 4. Two-way PERMANOVA confirmed that time point and soil treatment had a significant interaction effect on bacterial community composition (Table S4: R^2^ = 0.15, *p* < 0.05). NS communities clustered together independent of time points, while dynamic shifts in bacterial communities from week 0 to 6 were observed in IS and AS.

**Figure 5:**
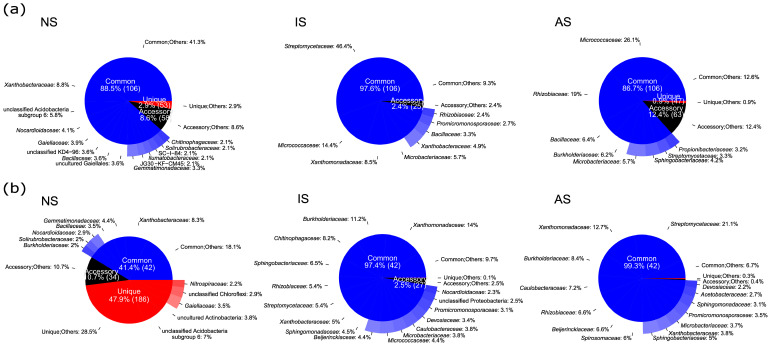
Mean relative abundance of bacterial families in different soil treatments (NS: natural soil, IS: inoculated soil, and AS: autoclaved soil) at two different time points (**a**: week 0 and **b**: week 6). Colors indicate three categories (blue as “Common”, black as “Accessory”, and red as “Unique”). Bacterial families found across all soil treatments are defined as “Common”, those unique to a soil are represented as “Unique”, with the remaining grouped as “Accessory”. Percentage shows the average relative abundance of each family (*n* = 3). “Common;Others”, “Unique;Others”, and “Accessory;Others” contain common/unique/accessory bacterial families that are less than 2% in relative abundance. The numbers in brackets show the number of bacterial families detected in each category.

The relative abundance of major bacterial families in NS, IS, and AS on weeks 0 and 6 is shown in Figure 5. Bacterial community in NS mainly included families assigned to *Xanthobacteraceae*, unclassified Acidobacteria subgroup 6, *Nocardioidaceae*, *Gaiellaceae*, unclassified KD4-96, *Bacillaceae*, uncultured Gaiellales, and *Gemmatimonadaceae*, irrespective of time points. On week 0, the major bacterial families in IS were *Streptomycetaceae*, *Micrococcaceae*, *Xanthomonadaceae*, *Microbacteriaceae*, *Xanthobacteraceae*, and *Bacillaceae*, while AS mainly consisted of *Micrococcaceae*, *Rhizobiaceae*, *Bacillaceae*, *Burkholderiaceae*, *Microbacteriaceae*, *Sphingobacteriaceae*, *Streptomycetaceae*, and *Propionibacteriaceae*. On week 6, IS were dominated by *Xanthomonadaceae*, *Burkholderiaceae*, *Chitinophagaceae*, *Sphingobacteriaceae*, *Rhizobiaceae*, *Streptomycetaceae*, and *Xanthobacteraceae*. AS consisted of *Streptomycetaceae*, *Xanthomonadaceae*, *Burkholderiaceae*, *Caulobacteraceae*, *Rhizobiaceae*, *Beijerinckiaceae*, *Spirosomaceae*, and *Sphingobacteriaceae* at the same time point. Overall, on week 0, 106 bacterial families were shared among all the soils, which represented 88.5%, 97.6%, and 86.7% of the total bacterial communities in NS, IS, and AS, respectively. Meanwhile, the number of unique bacterial families, which were only detectable in one of the soil treatments, corresponded to 2.9%, 0.03%, and 0.9% of the total bacterial communities in the same soil treatments. In contrast, on week 6, only 42 bacterial families were detected in all soil treatments, accounting for 41.4%, 97.4%, and 99.3% of the total bacterial communities in NS, IS, and AS, respectively. This was a consequence of the large number of unique bacterial families, detected only in NS and accounted for 47.9% of the bacterial community in the same soil.

Differences in the relative abundance of the dominant families among soil treatments and time points are shown in Figures S5 and S6. Independent of time points, the relative abundance of unclassified Acidobacteria subgroup 6, *Gaiellaceae*, *Gemmatimonadaceae*, *Ilumatobacteraceae*, JG30-KF-CM45, *Nitrospiraceae*, SC-I-84, *Solirubrobacteraceae*, unclassified KD4-96, and uncultured Gaiellales was significantly higher in NS than in IS and AS, while the relative abundance of *Acetobacteraceae* and *Rhizobiaceae* were higher in AS than in NS (*p* < 0.05 for each family, LS means test). The relative abundance of *Streptomycetaceae* was higher in IS than in NS and AS on week 0 (*p* < 0.05, LS means test). *Streptomycetaceae* significantly decreased in IS and increased in AS on week 6 (*p* < 0.05 for each soil treatment, LS means test), resulting in no significant differences in the relative abundance of *Streptomycetaceae* among the soil treatments on week 6. In addition, the relative abundance of *Nocardioidaceae*, *Sphingomonadaceae*, *Beijerinckiaceae*, *Devosiaceae*, and *Caulobacteraceae* significantly increased in both IS and AS from week 0 to 6 whereas *Spirosomaceae* increased only in AS during the same period. As a result, *Nocardioidaceae* and *Devosiaceae* were more enriched in IS than in AS on week 6, while the relative abundance of *Caulobacteraceae* and *Spirosomaceae* were higher in AS than in IS at the same time point (*p* < 0.05 for each family, LS means test).

#### 3.3.2. Maize Leaf Bacteria

**Figure 6:**
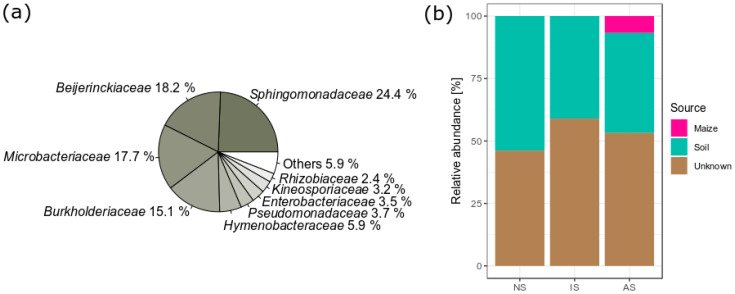
(**a**) Mean relative abundance of bacterial families in natural maize litter prior to soil application (*n* = 3). “Others” denotes bacterial families with less than 2% relative abundance. (**b**) The proportion of amplicon sequence variants (ASVs) in soil bacterial communities on week 6, which are estimated to come from different source environments using SourceTracker (*n* = 3). Maize and soil samples (NS: natural soil, IS: inoculated soil, and AS: autoclaved soil) on week 0 were considered to be source samples, while soils on week 6 were considered to be sink samples. ASVs that did not match a specific source environment were marked “Unknown”.

Bacterial communities that had colonized the natural maize litter prior to being applied to soils included *Sphingomonadaceae*, *Beijerinckiaceae*, *Microbacteriaceae*, *Burkholderiaceae*, and *Hymenobacteraceae* (Figure 6a). The bacteria derived from the maize litter increased only in AS, accounting for 6.78% of the bacterial community (Figure 6b). The ASVs derived from the maize litter were assigned to *Acetobacteraceae* (4 ASVs), *Beijerinckiaceae* (7 ASVs), *Burkholderiaceae* (3 ASVs), *Rhizobiaceae* (2 ASVs), *Sphingomonadaceae* (4 ASVs) *Caulobacteraceae* (1 ASV), *Microbacteriaceae* (1 ASV), *Nocardiaceae* (1 ASV), *Pseudomonadaceae* (1 ASV), and *Spirosomaceae* (1 ASV) (Table S5). The largest proportion of ASVs in NS, IS, and AS, however, was from soil and unknown sources.

## 4. Discussion

### 4.1. Manipulating Soil Bacterial Diversity

In this study, we aimed to examine the correlation between reduced bacterial diversity and maize litter decomposition rates, and to identify important bacterial taxa that gained a growth advantage 6 weeks after litter application. Furthermore, we investigated the extent to which litter-associated bacteria are capable of colonizing soil environments. In order to manipulate soil microbial diversity, a proportion of NS was autoclaved and reinoculated with microbes that were extracted from NS. In line with previous studies [44,65], we introduced significant shifts in the soil bacterial communities by autoclaving.

After preincubation, significant differences in bacterial community composition were evident between NS, IS, and AS, while bacterial abundance was similar between the three treatments. The presence of bacteria in AS could be explained by indigenous bacteria, which survived the autoclaving process and made use of nutrients derived from dead biomass and dissolved organic C (DOC) released by autoclaving [66]. The major ASVs in AS were assigned to Actinobacteria (*Streptomycetaceae*, *Micrococcaceae*, and *Microbacteriaceae*). Some actinobacterial strains are known to form spores and cyst-like cells, which could help them survive under harsh environmental conditions [66,67]. In addition, the ability to acquire C through aromatic compound and polysaccharide decomposition has been reported [68,69,70]. Such compounds are typically part of microbial necromass and thus may provide nutrients for recolonizing bacteria post-autoclaving. Besides, IS and AS consisted of *Xanthomonadaceae*, *Burkholderiaceae*, and *Sphingobacteriaceae*, which also have been described as bacterial groups capable of quickly recovering after a disturbance [71,72].

### 4.2. Bacterial Recovery after Disturbance

Several authors have postulated that a reduction in bacterial diversity is closely linked to reduced decomposition rates of plant residues. For example, Baumann et al. used γ-radiation for sterilization and reinoculation of sterilized soils with diluted soil suspensions to study the degradation of wheat-derived sugars [30]. As expected, they found that bacterial diversity positively affected sugar degradation in their study. In a similar setup, Maron et al. demonstrated that the cumulative soil respiration triggered by the application of wheat residues was significantly higher in soil with higher richness [42]. In line with these studies, we found that the rate of litter decomposition was higher in soils with higher bacterial diversity. In addition, we also found that the highest litter decomposition rate was accompanied by a stable bacterial community composition in NS, while the less diverse communities in IS and AS changed significantly over time. This adaptation of the bacterial communities in IS and AS might have caused delayed litter decomposition. Our results were in accordance with the theory that a high microbial diversity stabilizes the environment against disturbances [31,73,74]. Therefore, our corresponding results are likely due to the highly diverse community in NS, which held a wide variety of bacteria with different functional potentials. As NS was obtained from a field frequently planted with maize, this natural community might have been particularly adapted to decompose maize litter, as noted by de Vries et al. [75].

In our study, however, differences in fungal biomass might also be associated with different litter decomposition rates in the soil. Indeed, soil fungi became dominant in IS and AS, as indicated by the higher mean ratios of ITS copies to 16S rRNA gene copies (1.07 and 1.22 for IS and AS, respectively) compared to the ratio in NS (0.84) on week 0. This could be explained by the improved persistence of spores against autoclaving and the absence of antagonistic microbes after soil autoclaving [76]. However, the reduced litter decomposition rates, together with the high abundance of fungi in AS and IS, indicated that the importance of soil fungi is less pronounced in the early stages of litter decomposition. This underlines findings from previous studies that fungi played a more important role after several months of litter decomposition [26,27].

### 4.3. Oligotrophic Bacteria and Litter Decomposition

Bacterial members, which were excluded from IS and AS, such as Acidobacteria (especially subgroup 6), *Gaiellaceae*, and *Nitrospiraceae*, might have played an important role in litter decomposition. A high share of Acidobacteria has been observed in agricultural soils [77,78,79]. They are known as a good competitor in oligotrophic environments [80,81] and able to decompose plant polysaccharides [82,83]. The abundance of Acidobacteria subgroup 6 was especially reported to be positively correlated with soil nutrient concentrations (N and P). This subgroup includes the family *Vicinamibacteraceae*, known for a wide range of substrate preferences including recalcitrant C (e.g., chitin) and labile C [82,84], thus being functionally versatile and important to maintain functions in a given soil. *Gaiellaceae* [85] and *Nitrospiraceae* [86] were also characterized by oligotrophic lifestyles, yet the role of these families in litter decomposition is largely unknown. However, Actinobacteria were recently described as generalists in litter decomposition and contributed to nutrient mobilization [87]. Oligotrophic bacteria might have more possibilities to use different substrates, as compared to fast-growing copiotrophic bacteria, which have less flexibility in fluctuating nutrient amounts and quality in the early litter decomposition phases. Potential roles of oligotrophic bacteria tended to be masked in a microbial community as they were outcompeted by copiotrophic bacteria when nutrient levels increased [88]. Thus, genome analysis of single cells, isolates, or enrichment cultures would help to better understand their roles in litter decomposition.

Copiotrophic bacteria, which became more abundant during the early decomposition phases of the maize leaf litter-derived C, dominated in IS and AS. For example, the group included the genus *Sphingomonas* within *Sphingomonadaceae* and *Methylobacterium* within *Beijerinckiaceae,* which have the capability of degrading cellulose and hemicellulose [89,90]. The genus *Nocardioides* within *Nocardioidaceae* can assimilate plant-derived C during litter decomposition processes [91]. Additionally, we detected the N-fixing genera *Brevundimonas* within *Caulobacteraceae* [92], *Sphingomonas* within *Sphingomonadaceae* [93], and *Methylobacterium* within *Beijerinckiaceae* [94]. These bacteria benefit from high C availability during litter decomposition, which fuels the process of N fixation [95]. In contrast, fixed N might serve as an important nutrient for plant litter decomposers. Members of the families *Caulobacteraceae* and *Sphingomonadaceae* harbor alkaline phosphatases [96], again underpinning the importance of bacteria that fuel litter decomposition with nutrients provided by IS and AS. As a result of litter application, *Streptomycetaceae* decreased in relative abundance in IS, which mainly contained the plant-degrading genus *Streptomyces* [97]. The relative decrease of this family could be attributed to increasing bacterial competition for nutrient acquisition during litter decomposition.

### 4.4. Litter-Associated Bacteria and Litter Decomposition

The majority of bacteria derived from the natural maize litter consisted of *Sphingomonadaceae*, *Beijerinckiaceae*, *Microbacteriaceae*, and *Burkholderiaceae*. This corresponded to previous studies showing the low diversity of maize leaf microbiomes, represented by *Sphingomonadaceae* and *Microbacteriaceae* [98,99]. In addition, our data demonstrated that the litter microbiome might play an important role in litter decomposition, but only when both soil and litter materials were colonized by microbiomes with high diversity. Surprisingly, litter-associated bacteria, such as *Methylobacterium* within *Beijerinckiaceae*, successfully colonized only in AS during litter decomposition. However, the abundance of indigenous soil bacteria was also low in AS, which could increase the probability of a successful invasion from litter associated bacteria [32,33]. The genus *Methylobacterium* has also been detected in soils and different plant tissues [100] and described as methylotrophic [101], which could increase plant biomass decomposition in soil [102]. However, in this study, the invasion of *Methylobacterium* did not result in higher decomposition rates in AS.

## 5. Conclusions

Our results demonstrate that litter decomposition rates of maize leaf litter were positively correlated with soil bacterial diversity, and that litter application caused a significant change in bacterial community composition when diversity was low. In this study, the bacterial community composition in NS, primarily composed of oligotrophic bacteria including Acidobacteria subgroup 6, *Nitrospiraceae*, and *Gaiellaceae*, remained consistent, whilst the litter decomposition rate in NS was higher than in other soil treatments. In IS and AS, oligotrophic bacteria were absent. Instead, copiotrophic bacteria and litter-associated bacteria benefited from low competition and increasing C substrates. Thus, the absence of oligotrophic bacteria might have created a gap in functional richness and caused delayed litter decomposition, which was compensated after a lag period by the fast-growth of copiotrophic bacteria.

As we used autoclaved soil and microbial inoculation during this study, it was not necessarily comparable to natural conditions. For example, it has been reported that autoclaving increases DOC content of any material. Thus, we can assume that the autoclaved litter material might have contained larger amounts of DOC compared to the natural litter material. The high DOC content in the autoclaved maize compared to the natural maize could counteract the effect of plant-associated microbiomes on litter decomposition rates. Another limitation of this study is a missing strategy to exclude fungi from the experimental setup or to include them in our analysis. Indeed, our manipulation approach also influenced fungal diversity. However, this is a problem of all studies where similar strategies have been used to manipulate microbial diversity and can be considered as a drawback of the approach. Other methods (e.g., selective inhibition of fungi by antibiotic compounds) are also critical, and in many cases, only specific microbial groups are influenced by the treatment, or an additional effect on bacteria cannot be excluded. We also have reason to believe that fungi may not be strongly involved in the initial decomposition of litter material in our study. Future studies should also include the analysis of fungal communities during late phases of litter decomposition, where fungi are the main drivers for the decomposition of plant-derived polymers. Furthermore, ^13^C labeled litter material might help to identify microorganisms, which directly use the litter material, and those, which only indirectly benefit from plant-derived C (e.g., as major drivers for food web structuring).

## Figures and Tables

**Table 1:** Alpha-diversity indices (Shannon–Weaver diversity index, Pielou’s evenness, and amplicon sequence variants (ASVs)) of the soil bacterial communities at different time points (week −2, 0, and 6). Values represent the mean ± the standard deviation of three replicates. NS, IS, and AS stand for natural soil, inoculated soil, and autoclaved soil. We performed a two-way ANOVA to investigate the interaction between time points and soil treatments on each diversity index, followed by Tukey’s HSD test for pairwise comparisons. No significant interactions between time points and soil treatments were determined for any of the indices. Different letters represent significantly different pairs among soil treatments when averaged across time points (*p* < 0.05).

		Week −2	Week 0	Week 6	Tukey’s HSD
Shannon–Weaver diversity index	NS	6.95 ± 0.18	6.47 ± 0.10	6.83 ± 0.10	a
	IS	4.92 ± 1.03	3.27 ± 0.34	4.72 ± 0.30	b
	AS	5.17 ± 0.77	3.33 ± 1.10	4.25 ± 0.35	b
Pielou’s evenness	NS	0.95 ± 0.00	0.94 ± 0.00	0.95 ± 0.00	a
	IS	0.74 ± 0.12	0.62 ± 0.12	0.81 ± 0.04	b
	AS	0.75 ± 0.09	0.60 ± 0.07	0.78 ± 0.03	b
Number of ASVs	NS	1477 ± 276	961 ± 99	1361 ± 154	a
	IS	802 ± 283	226 ± 129	336 ± 41	b
	AS	997 ± 168	346 ± 254	228 ± 52	b

## Data Availability

The raw sequencing data presented in this study are openly available in in the NCBI Sequence Read Archive under the accession number SRP127524. All other data presented in this study are available in this article and the respective Supplementary Materials.

## Supplementary Materials

The following are available online at https://www.mdpi.com/2076-2607/9/2/357/s1, Figure S1: Results of 16S rRNA gene copy number quantification and alpha-diversity index (Shannon–Weaver diversity index and Pielou’s evenness) calculation based on qPCR and T-RFLP profiles during the preliminary test, Figure S2: Percentage of litter mass loss of natural and autoclaved maize litter in soils after 6 weeks of the incubation (NS: natural soil, IS: inoculated soil, and AS: autoclaved soil), Figure S3: Rarefaction curves of the number of observed amplicon sequence variants (ASVs) in the soil (NS: natural soil, IS: inoculated soil, and AS: autoclaved soil) and natural maize samples (*n* = 3), Figure S4: Scatter plots and regression lines showing the correlation between litter mass loss after 6 weeks and the alpha-diversity indices (Pielou’s evenness and the number of ASVs on week −2 (**a**,**d**), week 0 (**b**,**e**), and week 6 (**c**,**f**)), Figure S5: Relative abundance of dominant bacterial families (average relative abundance >2%) at different time points (weeks 0 and 6) and among soil treatments (NS: natural soil, IS: inoculated soil, and AS: autoclaved soil), Figure S6: Fold changes in the relative abundance of bacterial families (average relative abundance >2%) in response to litter addition, Table S1: RNA concentration in the natural soil (NS), inoculated soil (IS), and autoclaved soil (AS) sampled on week −2 (*n* = 3), Table S2: Primer sequences used for qPCR analysis and sequencing, Table S3: Reads obtained from the 16S rRNA gene sequencing, Table S4: Results of two-way PERMANOVA on Bray–Curtis dissimilarities of soil bacterial communities in different soil treatments (NS: natural soil, IS: inoculated soil, and AS: autoclaved soil) sampled at different time points (weeks 0 and 6), Table S5: List of the names and the relative abundance of maize-associated ASVs that were detected in autoclaved soil (AS) on week 6, Text S1 Experimental design for the preliminary test.
