# Supplementary material for: Soil Bacterial Diversity Is Positively Correlated with Decomposition Rates during Early Phases of Maize Litter Decomposition
Source: Microorganisms. 2021 Feb 11;9(2):357. doi: 10.3390/microorganisms9020357 (PMC7916959; doi:10.3390/microorganisms9020357)
Supplement: Supplementary file 1 [file microorganisms-09-00357-s001.pdf]

**Supplementary Materials**

Soil bacterial diversity is positively correlated with decomposition rates during early phases of maize litter decomposition

Authors

Akane Chiba[1,2,3], Yoshitaka Uchida[1], Susanne Kublik[2], Gisle Vestergaard[2,4], Franz Buegger[5], Michael Schloter[2,6], Stefanie Schulz[2*]

Affiliation:

[1] Research Faculty of Agriculture, Hokkaido University, Sapporo, Japan

[2] Research Unit Comparative Microbiome Analysis, Helmholtz Zentrum München, German Research Centre for Environmental Health, Neuherberg, Germany

[3] Crop Physiology, Technical University of Munich, Freising, Germany

[4] Section for Bioinformatics, Department of Health Technology, Technical University of Denmark, Denmark

[5] Institute of Biochemical Plant Pathology, Helmholtz Zentrum München, German Research Centre for Environmental Health, Neuherberg, Germany

[6] Chair of Soil Science, Technical University of Munich, Freising, Germany

*Corresponding author

(a)

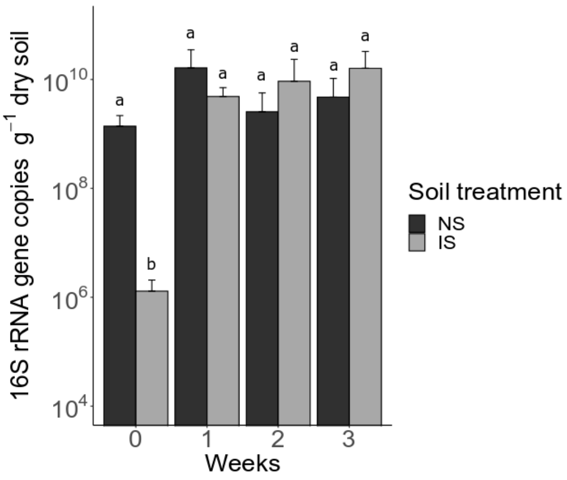


(b)

| | | Week 0 | Week 1 | Week 2 | Week 3 |
|---|---|---|---|---|---|
| Shannon-Weaver Diversity Index | NS | 3.27 ± 0.15 | 3.23 ± 0.09 | 2.90 ± 0.09 | 2.05 ± 0.68 |
| | | * | * | * | * |
| | IS | 2.68 ± 0.10 | 0.95 ± 0.04 | 1.75 ± 0.52 | 2.28 ± 0.10 |
| Pielou's evenness | NS | 0.87 ± 0.01 | 0.95 ± 0.03 | 0.97 ± 0.01 | 0.97 ± 0.02 |
| | | * | * | * | * |
| | IS | 0.67 ± 0.01 | 0.35 ± 0.02 | 0.48 ± 0.13 | 0.70 ± 0.03 |

**Figure S1.** Results of 16S rRNA gene copy number quantification and alpha-diversity index (Shannon-Weaver Diversity Index and Pielou's evenness) calculation based on qPCR and T-RFLP profiles during the preliminary test. (a) Copy numbers (mean + standard deviation of three replicates) of the 16S rRNA gene representing bacterial abundance in the natural soil (NS) and the inoculated soil (IS) during 3 weeks of the preliminary test. Samples were taken every week (week 0, 1, 2, and 3). Black and gray colors represent NS and IS, respectively. Bacterial abundance was log-transformed before statistical tests to improve the normality of the data distribution. We performed a two-way ANOVA to investigate the interaction between the time point and soil treatment on bacterial abundance, followed by Tukey's HSD test for pairwise comparisons. Different letters indicate significant differences ($p<0.05$) according to Tukey's HSD test. (b) Alpha-diversity indices (Shannon-Weaver Diversity Index and Pielou's evenness) of the soil bacterial communities based on T-RFLP profiles in NS and IS during the preliminary test. Values represent the mean ± the standard deviation of three replicates. We performed a two-way ANOVA to investigate the interaction between the time point and soil treatment on each diversity index, followed by Tukey's HSD test for pairwise comparisons. Asterisks indicate significant differences ($p<0.05$) according to Tukey's HSD test.

.

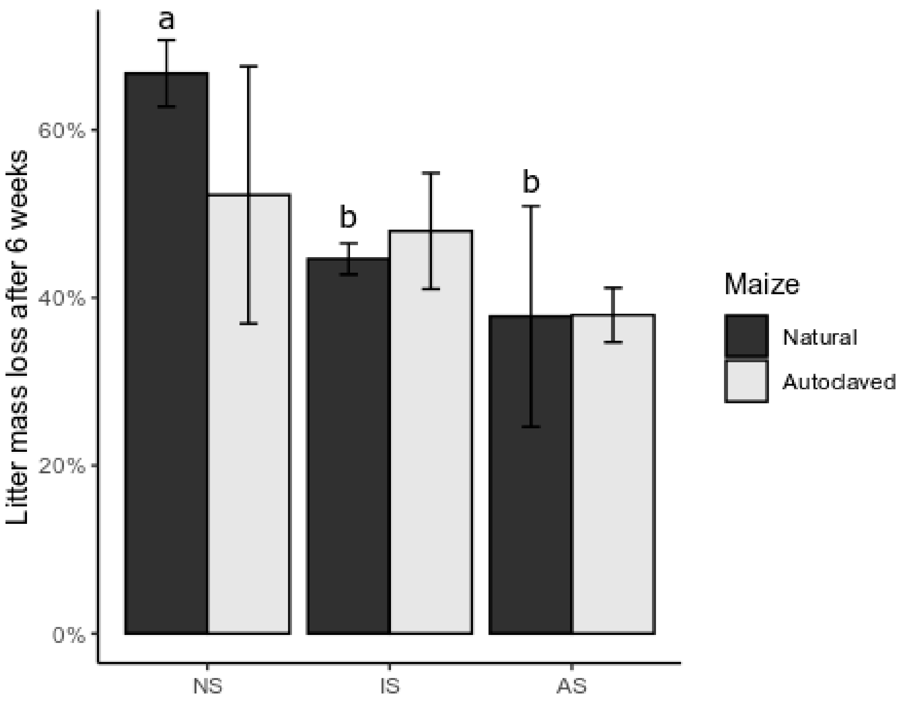


**Figure S2.** Percentage of litter mass loss of natural and autoclaved maize litter in soils after 6 weeks of incubation (NS: natural soil, IS: inoculated soil, and AS: autoclaved soil). Shown are mean ± standard deviation of three replicates. We performed a one-way ANOVA to investigate the effect of maize treatments and soil treatments on the mass loss of natural and autoclaved maize litter after 6 weeks, followed by the pairwise t-test and the Benjamini-Hochberg adjustment of p-values. No significant effect of maize treatments was determined. Different letters indicate significant differences ($p<0.05$) according to the pairwise t-test.

.

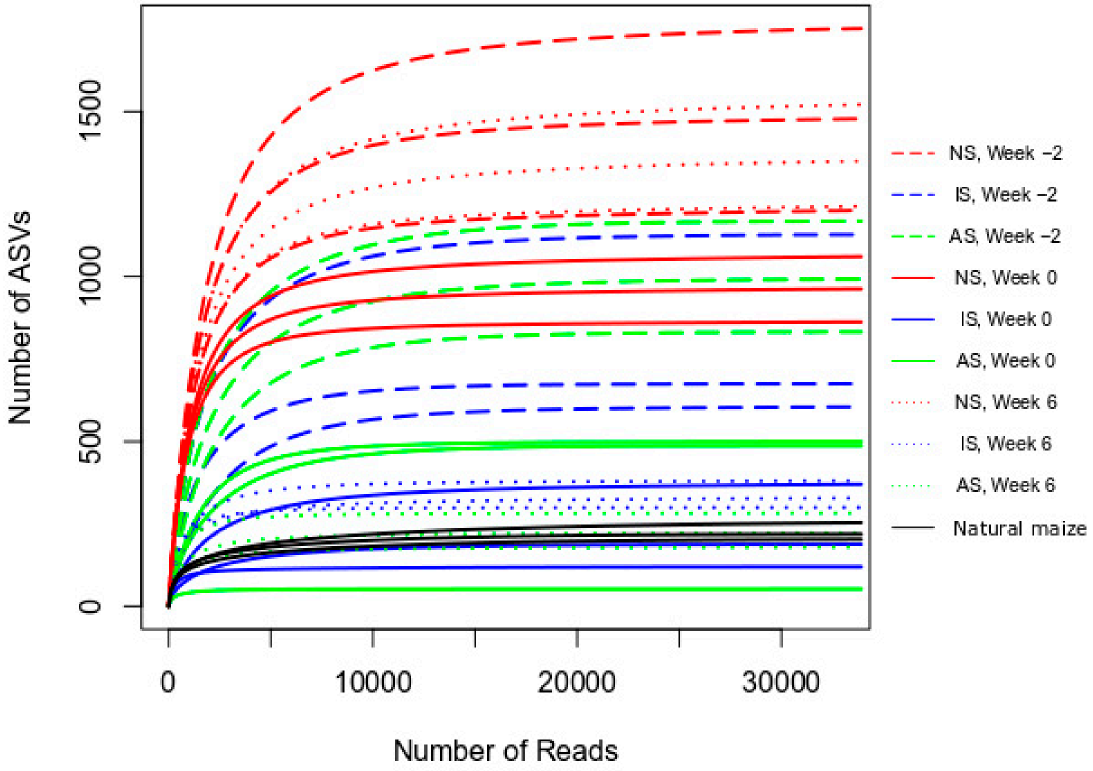


**Figure S3.** Rarefaction curves of the number of observed amplicon sequence variants (ASVs) in soil (NS: natural soil, IS: inoculated soil, and AS: autoclaved soil) and natural maize samples (n=3). Soil samples were analyzed at different time points (week –2, 0, and 6), while natural maize litter prior to litter application was analyzed. The vertical and horizontal axes represent the number of ASVs and sequence reads, respectively. Red, blue, green, and black colors stand for NS, IS, AS, and natural maize litter, respectively. Long dash, solid, and dotted lines represent week –2, 0, and 6, respectively, for the soil samples. .

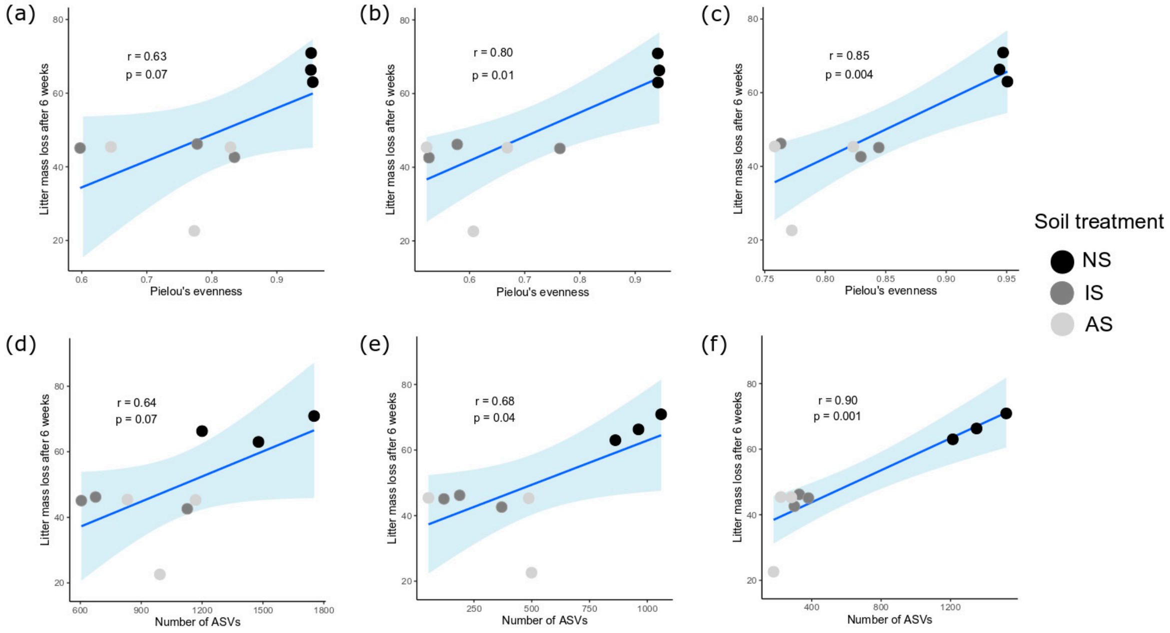


**Figure S4.** Scatter plots and regression lines showing the correlation between litter mass loss after 6 weeks and the alpha-diversity indices (Pielou's evenness and the number of ASVs on week -2 (a and d), week 0 (b and e), and week 6 (c and f)). Additionally, the 95% confidence interval, the Pearson's correlation coefficient (r), and p-value (p) are displayed. Black, gray and light gray dots indicate different soil treatments (NS: natural soil, IS: inoculated soil, and AS: autoclaved soil, n=3).

.

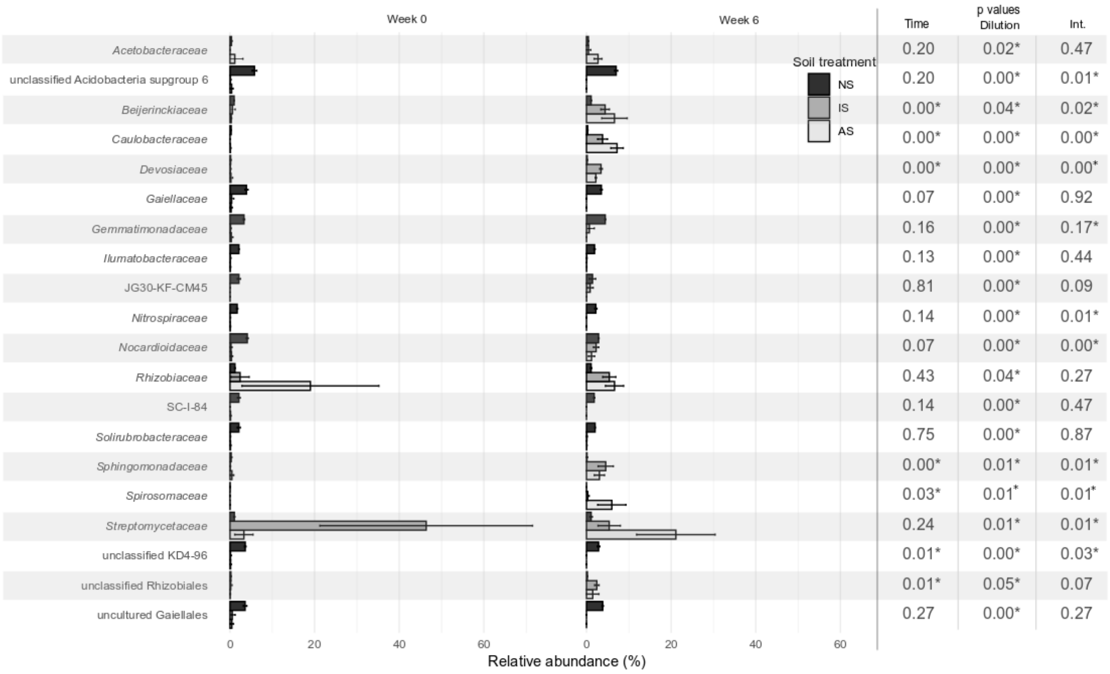


**Figure S5.** Relative abundance of dominant bacterial families (average relative abundance >2%) at different time points (week 0 and 6) and among soil treatments (NS: natural soil, IS: inoculated soil, and AS: autoclaved soil). Shown are mean values ± standard deviation of three replicates. The vertical and horizontal axes represent dominant bacterial families and their relative abundance, respectively. Black, gray, and white colors indicate NS, IS, and AS, respectively. Values on the right-hand side show p-values of the two-way ANOVA, followed by Benjamini-Hochberg adjustment. Asterisks indicate significant differences (p<0.05) according to Tukey's HSD test.

.

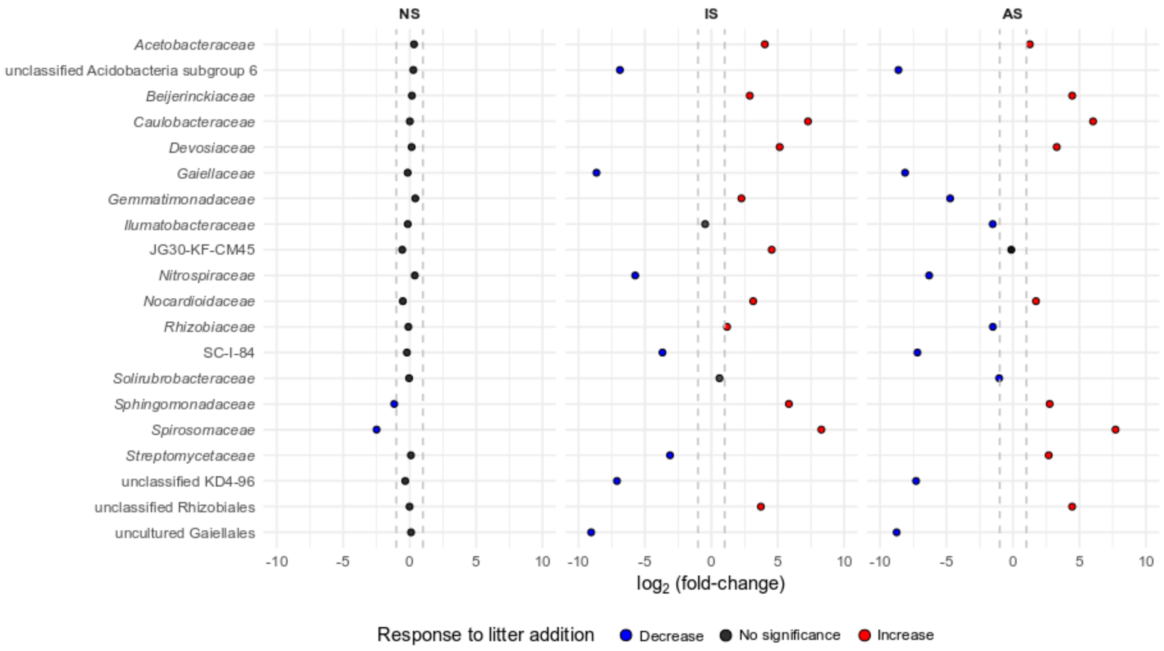


**Figure S6.** Fold changes in the relative abundance of dominant bacterial families (average relative abundance >2%) in response to litter addition. The fold changes (log2) were calculated by dividing the mean relative abundance of three replicates on week 6 by that on week 0. The vertical and horizontal axes represent dominant bacterial families and their fold-changes in soils (NS: natural soil, IS: inoculated soil, and AS: autoclaved soil), respectively. Two vertical dashed lines show a threshold of fold changes (fold changes of –2 and +2). Red and blue dots indicate the bacterial families with significantly higher and lower relative abundance on week 6 than week 0.

**Table S1.** RNA concentration in the natural soil (NS), inoculated soil (IS), and autoclaved soil (AS) sampled on week –2 (n=3).

| Soil | Replicate | RNA concentration (ng $g^{-1}$ dry soil) |
|---|---|---|
| NS | 1 | 59.88 |
| NS | 2 | 72.71 |
| NS | 3 | 94.14 |
| IS | 1 | 0.00 |
| IS | 2 | 0.28 |
| IS | 3 | 0.10 |
| AS | 1 | 0.10 |
| AS | 2 | 0.20 |
| AS | 3 | 0.29 |

**Table S2.** Primer sequences used for qPCR analysis and sequencing.

| Target gene | Primer | Sequence | Reference |
|---|---|---|---|
| 16S rRNA gene for qPCR | FP 16S | 5′-GGTAGTCYAYGCMSTAAACG-3′ | Bach *et al.* (2002)[48] |
| | RP 16S | 5′-GACARCCATGCASCACCTG-3′ | |
| ITS for qPCR | ITS 1 | 5′-TCCGTAGGTGAACCTGCGG-3′ | White *et al.* (1990)[49] |
| | ITS 4 | 5′-TCCTCCGCTTATTGATATGC-3′ | |
| 16S rRNA gene for sequencing | S-D-Bact-0008-a-S-16 | 5′-AGAGTTTGATCMTGGC-3′ | Klindworth *et al.* (2013)[51] |
| | S-D-Bact-0343-a-A-15 | 5′-CTGCTGCCTYCCGTA-3′ | |

**Table S3.** Reads obtained from the 16S rRNA gene sequencing. Values in the table show the number of raw reads and reads after quality filtering, denoising, merging, removing chimeric sequences, sequences assigned to chloroplasts at the order level, and singletons for the natural soil (NS), inoculated soil (IS), and autoclaved soil (AS) sampled on week –2, 0 and 6, and natural maize litter prior to being applied to the soils (n=3).

| Sample | Replicate | Raw reads | Filtered reads | Denoised reads | Merged reads | Non-chimeric reads | Reads without chloroplast | Reads without singletons |
|---|---|---|---|---|---|---|---|---|
| NS, Week –2 | 1 | 232140 | 138656 | 138656 | 88261 | 76114 | 75951 | 75939 |
| NS, Week –2 | 2 | 146807 | 92550 | 92550 | 56530 | 49764 | 49631 | 49624 |
| NS, Week –2 | 3 | 188784 | 114699 | 114699 | 71817 | 62604 | 62467 | 62452 |
| IS, Week –2 | 1 | 86759 | 52593 | 52593 | 42269 | 33942 | 33915 | 33899 |
| IS, Week –2 | 2 | 122420 | 72327 | 72327 | 54521 | 46867 | 46653 | 46643 |
| IS, Week –2 | 3 | 96466 | 62239 | 62239 | 56420 | 45506 | 45108 | 45098 |
| AS, Week –2 | 1 | 115802 | 71973 | 71973 | 55666 | 47595 | 47009 | 46999 |
| AS, Week –2 | 2 | 109602 | 66020 | 66020 | 56580 | 50803 | 48105 | 48099 |
| AS, Week –2 | 3 | 128681 | 78522 | 78522 | 59078 | 49400 | 49142 | 49127 |
| NS, Week 0 | 1 | 142445 | 83559 | 83559 | 48978 | 43324 | 43144 | 43137 |
| NS, Week 0 | 2 | 122848 | 77227 | 77227 | 45193 | 40061 | 40015 | 40006 |
| NS, Week 0 | 3 | 115771 | 68942 | 68942 | 40310 | 35624 | 35546 | 35530 |
| IS, Week 0 | 1 | 108354 | 70021 | 70021 | 64492 | 48386 | 48356 | 48352 |
| IS, Week 0 | 2 | 162905 | 96094 | 96094 | 84831 | 63763 | 63757 | 63733 |
| IS, Week 0 | 3 | 148410 | 94112 | 94112 | 86103 | 46922 | 46922 | 46922 |
| AS, Week 0 | 1 | 94740 | 62945 | 62945 | 55248 | 44017 | 43649 | 43584 |
| AS, Week 0 | 2 | 87171 | 56144 | 56144 | 54618 | 40438 | 40438 | 40438 |
| AS, Week 0 | 3 | 91505 | 60730 | 60730 | 54309 | 46882 | 46558 | 46555 |
| NS, Week 6 | 1 | 189032 | 118536 | 118536 | 73905 | 63934 | 63764 | 63740 |
| NS, Week 6 | 2 | 176213 | 104059 | 104059 | 63856 | 55041 | 54870 | 54851 |
| NS, Week 6 | 3 | 153532 | 94737 | 94737 | 57891 | 50426 | 50274 | 50266 |
| IS, Week 6 | 1 | 144396 | 85617 | 85617 | 78169 | 57042 | 57042 | 57005 |
| IS, Week 6 | 2 | 114896 | 71590 | 71590 | 62358 | 42167 | 42167 | 42167 |
| IS, Week 6 | 3 | 220828 | 139467 | 139467 | 115422 | 73226 | 73226 | 73226 |
| AS, Week 6 | 1 | 170861 | 100926 | 100926 | 92467 | 59478 | 59478 | 59460 |
| AS, Week 6 | 2 | 116606 | 71518 | 71518 | 65169 | 46576 | 46576 | 46536 |
| AS, Week 6 | 3 | 105837 | 62614 | 62614 | 55070 | 37694 | 37694 | 37679 |
| Fresh maize | 1 | 190862 | 109485 | 109485 | 99205 | 69270 | 44126 | 43334 |
| Fresh maize | 2 | 146706 | 89301 | 89301 | 80606 | 59394 | 35144 | 34676 |
| Fresh maize | 3 | 192623 | 112238 | 112238 | 101933 | 71469 | 53644 | 52881 |

**Table S4** Results of two-way PERMANOVA on the Bray-Curtis dissimilarities of soil bacterial communities in different soil treatments (NS: natural soil, IS: inoculated soil, and AS: autoclaved soil) sampled on different time points (week 0 and 6).

| PERMANOVA. | | |
|---|---|---|
| | $R^2$ | P-value |
| **Soil treatments** | 0.37 | <0.01 |
| **Time points** | 0.09 | 0.01 |
| **Soil treatments*Time points** | 0.15 | <0.01 |

**Table S5** List of the names and the relative abundance of maize-associated ASVs that were detected in autoclaved soil (AS) on week 6

| | Phylum | Family | Genus | Relative abundance % | | |
|---|---|---|---|---|---|---|
| ASV1 | Actinobacteria | *Microbacteriaceae* | *Microbacterium* | 0.03 | ± | 0.06 |
| ASV2 | Actinobacteria | *Nocardiaceae* | *Rhodococcus* | 0.02 | ± | 0.03 |
| ASV3 | Bacteroidetes | *Spirosomaceae* | *Larkinella* | 0.02 | ± | 0.03 |
| ASV4 | Proteobacteria | *Acetobacteraceae* | *Roseomonas* | 0.03 | ± | 0.06 |
| ASV5 | Proteobacteria | *Acetobacteraceae* | *Roseomonas* | 0.02 | ± | 0.03 |
| ASV6 | Proteobacteria | *Acetobacteraceae* | *Roseomonas* | 0.65 | ± | 0.41 |
| ASV7 | Proteobacteria | *Acetobacteraceae* | *Roseomonas* | 0.62 | ± | 0.45 |
| ASV8 | Proteobacteria | *Beijerinckiaceae* | *Methylobacterium* | 0.25 | ± | 0.31 |
| ASV9 | Proteobacteria | *Beijerinckiaceae* | *Methylobacterium* | 0.27 | ± | 0.38 |
| ASV10 | Proteobacteria | *Beijerinckiaceae* | *Methylobacterium* | 0.37 | ± | 0.15 |
| ASV11 | Proteobacteria | *Beijerinckiaceae* | *Methylobacterium* | 0.07 | ± | 0.12 |
| ASV12 | Proteobacteria | *Beijerinckiaceae* | *Methylobacterium* | 0.13 | ± | 0.08 |
| ASV13 | Proteobacteria | *Beijerinckiaceae* | *Methylobacterium* | 0.15 | ± | 0.26 |
| ASV14 | Proteobacteria | *Beijerinckiaceae* | *Methylobacterium* | 1.65 | ± | 2.51 |
| ASV15 | Proteobacteria | *Burkholderiaceae* | *Variovorax* | 0.13 | ± | 0.03 |
| ASV16 | Proteobacteria | *Burkholderiaceae* | *Variovorax* | 0.03 | ± | 0.06 |
| ASV17 | Proteobacteria | *Burkholderiaceae* | *Massilia* | 0.30 | ± | 0.10 |
| ASV18 | Proteobacteria | *Caulobacteraceae* | *Brevundimonas* | 0.18 | ± | 0.16 |
| ASV19 | Proteobacteria | *Pseudomonadaceae* | *Pseudomonas* | 0.03 | ± | 0.06 |
| ASV20 | Proteobacteria | *Rhizobiaceae* | *Aureimonas* | 0.12 | ± | 0.16 |
| ASV21 | Proteobacteria | *Rhizobiaceae* | *Allorhizobium-Neorhizobium-Pararhizobium-Rhizobium* | 0.47 | ± | 0.46 |
| ASV22 | Proteobacteria | *Sphingomonadaceae* | *Sphingomonas* | 0.35 | ± | 0.33 |
| ASV23 | Proteobacteria | *Sphingomonadaceae* | *Sphingomonas* | 0.47 | ± | 0.72 |
| ASV24 | Proteobacteria | *Sphingomonadaceae* | *Sphingomonas* | 0.10 | ± | 0.17 |
| ASV25 | Proteobacteria | *Sphingomonadaceae* | *Sphingomonas* | 0.33 | ± | 0.49 |
| | SUM | | | 6.78% | | |

**Text S1** Experimental design for the preliminary test

To determine the length of the pre-incubation period between microbial inoculation and litter addition, an incubation experiment with the natural soil (NS) and inoculated soil (IS) was carried out. First, 21 g of NS and IS were placed in Falcon™ 50 mL conical centrifuge tubes with open headspace. Under the same experimental conditions of soil moisture and room temperature as in the main text, the soils were incubated for 3 weeks. Soil samples were sampled right after the addition of the inoculum (week 0) and each of the following weeks, and stored at –20 °C for molecular analysis. For each treatment and time point, 3 parallel tubes were prepared and treated as true replicates throughout the preliminary test. Genomic DNA was extracted from 0.30 g of soil using the NucleoSpin®Soil Kit (Macherey-Nagel, Germany) following the manufacture's instruction. Soil bacterial abundance was quantified using a SYBR Green-based quantitative real-time PCR (qPCR) assay on a 7300 Real-time PCR System (Applied Biosystems, USA) as described in the main text. The optimal dilution rate of DNA extracts was 1:32 for soils. Missing values under detection limit (one sample for IS on week 2 and another sample for IS on week 3) were replaced by the values obtained with 1:64 dilution. Soil bacterial diversity was assessed using terminal restriction fragment length polymorphism (T-RFLP) analysis based on 16S rRNA gene using the primers 27f (FAM - labelled) and 1401r following the instruction by Gschwendtner et al. [100]. Briefly, PCR runs began at 94 °C for 5 min, followed by 30 cycles of amplification (94 °C for 1 min, 55 °C for 1 min, and 72 °C for 1 min), and the final extension at 72 °C for 10 min. The reaction was performed in a total volume of 50 µl, containing 1×Taq buffer (Life Technologies), 2.5 mM of $MgCl_2$ (Fermentas, St. Leon Rot, Germany), 0.2 mM of dNTPs (Fermentas), 0.2 µM of each primer (Metabion), 150 µg of BSA (Sigma Aldrich), 2.5 U of Taq Polymerase (Life Technologies) and 20 ng of template DNA. The amplicons were purified using NucleoSpin®Gel and PCR Clean-up kit (Macherey Nagel, Germany) and digested with the restriction enzyme MspI (Fermentas) at 37 °C for 4 hours. The enzymatic reaction was stopped by incubating at 80 °C for 20 min. The digested amplicons were purified using NucleoSpin®Gel and PCR Clean-up kit and quantified using the Quant-iT PicoGreen dsDNA Assay Kit (Invitrogen, USA). The terminal restriction fragments (T-RFs) were separated based on their size by an ABI 3730 sequencer using MapMarker 1000 (Eurogentec, Köln, Germany) as an internal standard [100]. Size and relative abundances of the T-RFs were analyzed using GeneMapper v4.0 software (Life Technologies) and the online software T-REX (http://trex.biohpc.org/) with a binning range of 2 bp. T-RFs smaller than 50 bp and/or smaller than 1% of the total peak height were excluded. The data analysis of the T-RFLP profiles was carried out with the statistical software R (version 3.5.3) by calculating

the relative abundance of T-RFs per soil sample. Alpha diversity indices were calculated using the diversity function in the vegan package.

**Reference**


48 Bach, H. J.; Tomanova, J.; Schloter, M.; et al. Enumeration of total bacteria and bacteria with genes for proteolytic activity in pure cultures and in environmental samples by quantitative PCR mediated amplification. *J. Microbiol. Methods* **2002,** 49, 235–245.

49 White, T. J.; Bruns, T.; Lee, S.; et al. Amplification and direct sequencing of fungal ribosomal RNA genes for phylogenetics. In *PCR protocols: a guide to methods and applications*. Innis, M., Gelfand, D., Sninsky, J., White, T., Eds.; Academic Press: New York, USA, 1990; pp. 315-322.

51 Klindworth, A.; Pruesse, E.; Schweer, T.; et al. Evaluation of general 16S ribosomal RNA gene PCR primers for classical and next-generation sequencing-based diversity studies. *Nucleic Acids Res.* **2013**, *41*, e1.

103 Gschwendtner, S; Leberecht, M.; Engel, M; et al., Effects of elevated atmospheric $CO_2$ on microbial community structure at the plant-soil interface of young beech trees (*Fagus sylvatica* L.) grown at two sites with contrasting climatic conditions. *Microb. Ecol.* **2015,** 69, 867–878.
